# Nurses’ Perceptions of Professional Practice Environment and Its Relation to Missed Nursing Care and Nurse Satisfaction

**DOI:** 10.3390/ijerph17113805

**Published:** 2020-05-27

**Authors:** Renáta Zeleníková, Darja Jarošová, Ilona Plevová, Eva Janíková

**Affiliations:** Department of Nursing and Midwifery, Faculty of Medicine, University of Ostrava, 703 00 Ostrava, Czech Republic; darja.jarosova@osu.cz (D.J.); ilona.plevova@osu.cz (I.P.); eva.janikova@osu.cz (E.J.)

**Keywords:** professional environment, missed care, nurse satisfaction

## Abstract

The professional practice environment is a factor that can have a significant impact on missed nursing care. The study aimed to find a relationship between nurses’ perceptions of their professional practice environment and missed nursing care and job satisfaction. An additional aim was to find differences in nurses’ perceived rating of the professional practice environment according to hospital location and job position. A descriptive correlational study was performed. The sample included 513 general and practical nurses providing direct care in nine Czech hospitals. The Revised Professional Practice Environment scale and the Missed Nursing Care (MISSCARE) survey were used to collect data. The professional practice environment was most correlated with satisfaction with the current position (0.4879). The overall score of missed care correlated most strongly with the subscale “staff relationships” (−0.2774). Statistically significant differences in the rating of two subscales, “control over practice” and “cultural sensitivity”, were found between nurses from hospitals in district capitals and those from hospitals in smaller cities. Statistically significant differences in the rating of the “leadership and autonomy in clinical practice” and “teamwork” subscales were found between general nurses and practical nurses. The professional practice environment is related to nurse satisfaction and missed nursing care.

## 1. Introduction

The professional practice environment is a factor that can have a significant impact on missed nursing care [1]. At the same time, it is a factor of great significance in the recruitment and retention of healthcare professionals [2]. Aspects of the professional practice environment directly and indirectly influence the quality of nursing care [2].

Several studies confirmed that nurses in hospitals with a favorable professional practice environment reported less missed care [3,4,5]. Research in 1406 hospitals across nine countries revealed that hospitals with consistently better professional practice environments have lower nurse burnout and job dissatisfaction, and that nurses have a more positive perception of the quality of care on their units [6]. The associations between an unfavorable professional practice environment and nurse dissatisfaction and burnout, and between an unfavorable professional practice environment and quality care deficits were also confirmed in other studies [7,8].

The professional practice environment is defined by Lake [9] as “the organizational characteristics of a work setting that facilitate or constrain professional nursing practice”. Professional practice environments support nurses to work at the highest scope of nursing practice, to work effectively in a multidisciplinary team, and to mobilize resources quickly [10]. According to the World Health Organization (WHO), “an attractive and supportive work environment can be described as an environment that attracts individuals into the health professions, encourages them to remain in the health-workforce, and enables them to perform effectively” [2]. Professional practice environment is, in the scientific literature, also known as work environment, working conditions, and job characteristics [11]. The professional practice environment of nurses is one of the predictors of missed nursing care [3,12]. The work conditions can be grouped in different concepts such as nurse participation, supportive managers, staffing, patient-centered climate, autonomy, philosophy focusing on quality of care, collaborative relationships with physicians, collaborative relationships with peers, decentralization, and busyness [11].

Several instruments were developed to measure the work environment, one of which is the Revised Professional Practice Environment (RPPE) scale, used in nursing research to evaluate nurses’ perception of the nursing work environment [13]. The RPPE scale is a multidimensional tool comprising eight components of professional clinical practice in acute care settings [14]. It was first published in 2009 and was since used in several studies across various settings, from acute care hospitals [15] to care settings for older people [16] and emergency departments [17]. However, although it was used in different settings and different countries, the RPPE scale was never used in the Czech Republic.

According to a report by Page [18], since the nursing workforce is one of the most important factors in providing safe patient care in the healthcare system, it is crucial to improve nurses’ work environment during times of staff scarcity. The report reveals that “the typical work environment of nurses is characterized by many serious threats to patient safety” [18]. There are several published studies on the topic worldwide, and interest in the topic in Central Europe is high due to lack of research in this area. In addition, the professional practice environment of nurses is increasingly coming to managers´ attention due to the shortage of nurses, the higher average age of Czech nurses, and the higher nurse/patient ratio.

Missed nursing care is defined as “any aspect of required patient care that is omitted (either in part or in whole) or significantly delayed” [19]. The Missed Nursing Care Model was chosen as a theoretical framework for this study [20,21]. According to the model, missed nursing care is predicted by staff characteristics, together with hospital and unit characteristics. The model predicts relationships between the practice environment, nursing process and patient outcomes. The Missed Nursing Care Model postulates many relationships, e.g., that missed nursing care predicts job satisfaction, that staffing type and teamwork predicts missed nursing care, and that size of hospital predicts teamwork [22]. Our study focused predominantly on the relationship between the work environment of Czech nurses and missed nursing care. Our hypothesis is that a positive professional practice environment would be associated with less missed nursing care and better job satisfaction. 

The study aimed to find a relationship between nurses’ perceptions of the professional practice environment and missed nursing care and nurse satisfaction. An additional aim was to find differences in nurses’ perceived rating of the professional practice environment according to hospital location and job position.

## 2. Materials and Methods

### 2.1. Design

A Descriptive Correlational Study.

### 2.2. Participants

The sample included 513 general nurses and practical nurses providing direct care in hospitals of various size and organizational structure located in the Moravian-Silesian Region of the Czech Republic. Nine hospitals agreed to participate in the study. The total nurse population of the Czech Republic is large, at over 80,000. The sample size was calculated as 382 nurses, giving the study a margin of error of ±5% (confidence interval 95%). From a total of 822 distributed questionnaires, 513 were completed (a response rate of 62.4%).

### 2.3. Data Collection

Data were collected from January to August 2019 using a paper-and-pencil questionnaire. To measure the main variables (perception of professional practice environment, and missed nursing care), the RPPE scale [14] and the Missed Nursing Care (MISSCARE) survey [20] were used. Both questionnaires were translated into Czech using forward–backward translation. The Czech version of the MISSCARE Survey was previously used and tested in the Czech Republic [23]. In addition, unit staff characteristics (age, gender, education, job position, job satisfaction) and hospital characteristics (hospital location) were examined. Hospitals where the nurses included in the study worked were assessed according to their location—either district capitals or smaller cities. Three hospitals were located in district capitals (more than 300 beds), and six hospitals were located in smaller cities (with fewer than 300 beds, bar one exception). As another key study variable, job position of nurses was examined. Two groups—general nurses and practice nurses—were involved in the study.

Job satisfaction is defined as a positive affective orientation toward employment [24]. To measure nurse job satisfaction, three single-item scales were used. Nurses were asked to rate their satisfaction with their current position, their satisfaction with being a nurse or a nurse assistant, and their satisfaction with the level of teamwork on a five-point Likert scale from 1—very satisfied to 5—very dissatisfied.

The RPPE is a 39-item scale. Nurses were asked to indicate their level of agreement on a four-point Likert-type scale ranging from 1—strongly disagree to 4—strongly agree. The scale consists of the following eight sub-scales: “leadership and autonomy in clinical practice” (five items), “control over practice” (five items), “communication about patients” (three items), “teamwork” (four items), “handling disagreements” (nine items), “staff relationships” (two items), “internal work motivation” (eight items), and “cultural sensitivity” (three items). Eight items required reversal. Item 22 (“staff withdraw from conflict”) was not reversed as recommended. Statistical analysis showed that Czech nurses understood item 22 in a way opposed to its original sense. 

The reliability of the entire RPPE scale in this study was 0.89. Cronbach’s alpha for the subscales was as follows: “leadership and autonomy in clinical practice”: 0.71, “control over practice”: 0.78, “communication about patients”: 0.60, “teamwork”: 0.59, “handling disagreements”: 0.71, “internal work motivation”: 0.80, and “cultural sensitivity”: 0.62. Factor analysis revealed eight factors.

The MISSCARE Survey comprises 24 items in part A (activities of nursing care) and 17 items in part B (reasons for missed care). In part A, nurses are asked to indicate the frequency with which care is missed using the scale “rarely”, “occasionally”, “frequently”, “always”, or “non-applicable”. In part B, nurses are asked to rate each item using the scale “significant factor”, “moderate factor”, “minor factor”, or “not a reason for unmet nursing care”.

In the present study, Cronbach’s alpha for parts A and B was 0.97 and 0.92, respectively. In this study, only total scores (the mean frequency ratings across all items) of part A were used.

The research protocol was approved by the institutional ethics committee (no. 1/2019 Ethics Committee, Faculty of Medicine, University of Ostrava, Czech Republic).

### 2.4. Data Analysis

After data cleaning, frequencies were calculated to explore the distribution of nurses’ perceptions of the professional practice environment, nurse satisfaction, missed care, staff, and hospital characteristics. Spearman’s correlation coefficient was used to test relationships between both missed care and nurse satisfaction and nurses’ perceptions of the professional practice environment. The Wilcoxon–Mann–Whitney two-sample rank-sum test was used to test differences in rating the professional practice environment by job position and hospital location. Data were analyzed using the Stata software package.

## 3. Results

The mean age of nurses was 38 (SD 11.1) years. The majority were general nurses (78%), female (97%), and graduates from secondary vocational schools (67%). Slightly more than half worked in hospitals in district capitals (51%). The majority of nurses were satisfied with their current position, with being a nurse, and with the level of teamwork on their units (Table 1).

The highest scores (3.0) were obtained for the subscales “control over practice”, “communication about patients”, and “staff relationships”. These subscales were rated as favorable. “Teamwork”, with the lowest mean (2.2), was the worst-rated subscale. The lowest rated item of the RPPE scale was “there are enough staff to provide quality patient care” (2.0) from the “control over practice” subscale. The highest rated item was “I feel a high degree of personal responsibility for the work I do” (3.4) from “internal work motivation” (Table 2).

There were statistically significant differences in the rating of two subscales, “control over practice” and “cultural sensitivity”, between nurses from hospitals in district capitals and those in smaller cities. Nurses from hospitals in district capitals rated “control over practice” significantly more highly than did nurses from smaller city hospitals. By contrast, nurses from smaller city hospitals rated “cultural sensitivity” significantly more highly. The total RPPE scale score was slightly higher among nurses from hospitals in district capitals (Table 3).

General nurses generally rated their professional practice environment (RPPE total score) slightly more highly than practical nurses. Statistically significant differences in the rating of the subscales “leadership and autonomy in clinical practice” and “teamwork” were found between general nurses and practical nurses. While “leadership and autonomy in clinical practice” was rated statistically significantly more highly by practical nurses, “teamwork” was rated statistically significantly more highly by general nurses (Table 4).

The overall score of missed nursing care negatively correlated with the RPPE total score (−0.2141), i.e., a better professional practice environment reflected a lower level of missed nursing care. This correlation was weak. The overall score of missed care correlated most strongly with the subscale “Staff relationships” (−0.2774). The correlation was negative, i.e., better staff relationships reflected a lower level of missed nursing care. The professional practice environment (RPPE total score) correlated most with satisfaction with current position (0.4879). Satisfaction with the level of teamwork on this unit correlated with the RPPE total score (Table 5).

## 4. Discussion

The professional practice environment is linked to different nurse outcomes such as unmet patient care needs, job satisfaction, burnout, intention to leave, and missed nursing care [6,25,26]. The present study investigated the professional practice environment of Czech nurses and its relation to missed care and nurse satisfaction. In this respect, the study is unique, since Czech nurses were not previously the focus of such research.

In the present study, the worst-rated item of the RPPE scale was “there are enough staff to provide quality patient care”. Staff shortage is a consistently reported problem in Czech samples in nursing research [23,27], as well as in other international studies [28,29,30,31]. However, despite this shortage, missed care may be reduced when a positive work environment for nurses is ensured. Several previous studies [28,32] suggested that teamwork and cooperation are associated with lower reports of missed care. In the present study, “teamwork”, containing items related to teamwork and communication with other departments, was the worst-rated subscale. A study by Kalisch and Lee [21] confirmed that, when teamwork was stronger, less missed nursing care was reported. In contrast, “staff relationships” was the most highly rated subscale in the present study. Relationships within and communication between departments may be improved by appropriate interventions.

Data analysis further revealed that nurses’ satisfaction with the level of teamwork on their units significantly correlated with the RPPE total score. Professional practice environment correlated most with satisfaction with current position. The association between the professional practice environment, resource adequacy, and nurse satisfaction was confirmed in a systematic review [33]. Nurses’ perceptions of their professional environment influence their job satisfaction [33].

It is well documented that nurse satisfaction has a significant impact on both nurses and patients [34]. Moreover, lower nurse satisfaction with current position leads to more frequent missed care [35]. In the present study, the most highly rated item of the RPPE scale was related to internal work motivation, suggesting this as the reason for the high level of nurse satisfaction.

The additional aim of this study was to find differences in nurses’ perceived rating of the professional practice environment according to hospital location and job position. Although the practice environment was rated slightly more highly by nurses from hospitals in district capitals than by those from hospitals in smaller cities, the differences were not statistically significant. Hospital location is probably a variable with only a minor impact on the professional practice environment. 

General nurses rated the professional environment generally slightly more highly than practical nurses. Differences were found for two subscales, “leadership and autonomy in clinical practice” and “teamwork”, with the former being rated more highly by practical nurses and the latter by general nurses.

Several studies confirmed that practice environment is statistically significantly related to missed nursing care. Better nursing practice environments are associated with less missed nursing care [4,5,36,37]. In a better practice environment, nurses miss approximately one fewer necessary care activity [37]. A recent study by Lake, Riman, and Sloane [12] indicated a reduction in missed care in hospitals with better work environments or improved nurse staffing, with the effect of changes in the work environment being greater than that of nurse staffing on missed care. The conclusion of a systematic review by Zhao et al. [1] also confirmed the negative correlation between the professional environment and missed care [1]. 

Kim, Yoo, and Seo [38] (p. 125) stated that “missed nursing care is not an outcome associated with individual skills, but an organizational quality of nursing affected by nursing work environment factors”. Improvements to the work environment may contribute to ensuring both the supply of a healthy workforce and the enhancement, effectiveness and motivation of that workforce [2].

### 4.1. Implication for Nursing Practice

Significant reductions in rationing nursing care may be achieved by identifying modifiable features of the nursing practice environment. The results of this study suggest the need for strategies to improve the work environment. The establishment of a positive practice environment is of importance in guaranteeing quality of care for patients.

### 4.2. Limitation

The selection of nurses from a single region in Czech Republic did not allow extrapolation of the results to the entire country. In addition, cross-sectional design and self-reporting data may be sources of potential biases. Despite these limitations, similar results from various previous studies tend to support the validity of our findings.

## 5. Conclusions

The professional practice environment is linked to nurse satisfaction, as well as missed nursing care. Missed nursing care in hospitals can be reduced by improvements to the professional practice environment, with an emphasis on strengthening teamwork. Better work environments will likely improve nurses’ satisfaction with their current position, as well as with the level of teamwork on their units.

## Figures and Tables

**Table 1:** Sample characteristics.

Characteristic		*n*	%
Gender (*n* = 498)	Female	482	97%
	Male	16	3%
Education (*n* = 497)	Secondary vocational school	331	67%
	Higher degree (diploma) or university	166	33%
Position at work (*n* = 507)	General nurse	395	78%
	Practical nurse	112	22%
Satisfaction with the current position (*n* = 510)	Very satisfied	66	13%
	Satisfied	277	54%
	Neutral	135	27%
	Dissatisfied	25	5%
	Very dissatisfied	3	1%
Satisfaction with being a nurse (*n* = 511)	Very satisfied	115	23%
	Satisfied	297	58%
	Neutral	78	15%
	Dissatisfied	16	3%
	Very dissatisfied	2	1%
Satisfaction with the level of teamwork on unit (*n*=510)	Very satisfied	64	12%
	Satisfied	279	55%
	Neutral	121	24%
	Dissatisfied	35	7%
	Very dissatisfied	9	2%
Hospital location (*n* = 513)	District capital	263	51%
	Small city	250	49%

**Table 2:** Nurses’ perception of the professional practice environment (*n* = 513). RPPE—Revised Professional Practice Environment.

	RPPE Subscale/Item	Mean	Median	SD	Min	Max
	**Leadership and Autonomy in Clinical Practice**	**2.8**	**2.78**	**0.35**	**1.44**	**3.89**
1	Leadership is supportive of my department/unit staff.	2.9	3.00	0.79	1	4
2	My discipline controls its own practice.	3.0	3.00	0.66	1	4
3	I have freedom to make important patient management and work decisions.	2.9	3.00	0.60	1	4
8	My unit/department head is a good manager and leader.	3.2	3.00	0.71	1	4
11	My unit/department head supports the staff in decision-making, even if the conflict is with a physician.	3.0	3.00	0.73	1	4
	**Control over Practice**	**3.0**	**3.00**	**0.48**	**1**	**4**
5	I have adequate support services to allow me to spend time with my patients.	2.1	2.00	0.72	1	4
6	I have enough time and opportunity to discuss patient management problems with other staff.	2.4	2.00	0.72	1	4
7	There are enough staff to provide quality patient care.	2.0	2.00	0.78	1	4
9	We have enough staff to get the work done.	2.1	2.00	0.79	1	4
10	There are opportunities to work on a highly specialized patient care unit.	2.5	3.00	0.78	1	4
	**Communication about Patients**	**3.0**	**3.00**	**0.43**	**1.63**	**4**
13	Information on the status of patients is available when I need it.	3.1	3.00	0.62	1	4
14	I receive information quickly when a patient’s status changes.	2.7	3.00	0.65	1	4
15	There are needless delays in relaying information about patient care. *	3.1	3.00	0.56	1	4
	**Teamwork**	**2.2**	**2.20**	**0.56**	**1**	**4**
16	My unit/department has constructive work relationships with other groups in this hospital.	2.7	3.00	0.59	1	4
17	My unit/department does not receive the cooperation it needs from other hospital units/departments. *	2.6	3.00	0.68	1	4
18	Other hospital units/departments seem to have a low opinion of my unit/department. *	2.4	2.00	0.83	1	4
19	Inadequate working relationships with other hospital groups limit the effectiveness of work on this unit. *	2.7	3.00	0.69	1	4
	**Handling Disagreements**	**2.6**	**2.50**	**0.47**	**1**	**4**
20	When staff disagree, they ignore the issue, pretending it will “go away”. *	2.9	3.00	0.67	1	4
21	Most conflicts occur with members of my own discipline.	2.8	3.00	0.68	1	4
22	Staff withdraw from conflict.	2.6	3.00	0.63	1	4
23	All points of view are carefully considered in arriving at the best solution for the problem.	2.9	3.00	0.57	1	4
24	All staff work hard to arrive at the best possible solution.	2.8	3.00	0.72	1	4
25	Staff involved in a disagreement or conflict do not settle the dispute until all are satisfied with the decision.	2.4	2.00	0.64	1	4
26	All contribute from their experience and expertise to produce a high quality solution for a conflict.	2.8	3.00	0.58	1	4
27	Disagreements between staff are ignored or avoided. *	2.8	3.00	0.62	1	4
28	Staff involved in a disagreement or conflict settle the dispute by consensus.	2.7	3.00	0.52	1	4
	**Staff Relationships**	**3.0**	**3.00**	**0.46**	**1.33**	**4**
4	There is a lot of teamwork between unit/department staff and doctors.	2.8	3.00	0.64	1	4
12	Physicians and staff have good working relationships.	2.8	3.00	0.60	1	4
	**Internal Work Motivation**	**2.8**	**3.00**	**0.42**	**1**	**4**
29	My opinion of myself goes up when I work in this unit/department.	2.5	3.00	0.73	1	4
30	I feel bad and unhappy when I discover that I have performed less well than I should.	2.9	3.00	0.69	1	4
31	I feel a high degree of personal responsibility for the work I do.	3.4	3.00	0.62	1	4
32	I feel a great sense of personal satisfaction when I do my work well.	3.3	3.00	0.62	1	4
33	I have challenging work that motivates me to do the best job I can.	3.2	3.00	0.61	1	4
34	Working in this unit/department gives me the opportunity to gain new knowledge and skills.	3.1	3.00	0.66	1	4
35	I am motivated to do well because I am empowered by my work environment.	2.8	3.00	0.68	1	4
36	Working in this environment increases my sense of professional growth.	2.7	3.00	0.67	1	4
	**Cultural Sensitivity**	**2.8**	**3.00**	**0.55**	**1**	**4**
37	Staff have access to the necessary resources to provide culturally competent care.	2.7	3.00	0.60	1	4
38	Staff are sensitive to the diverse patient population for whom they care.	2.9	3.00	0.56	1	4
39	Staff respect the diversity of their health care team.	2.9	3.00	0.51	1	4

* Reversed item. Note: Subscale in bold.

**Table 3:** Differences in rating of the professional practice environment according to hospital location.

RPPE Scale Domain	Hospital Location	*n*	Median	Mean	SD	Min	Max	*p*-Value
Leadership and autonomy in clinical practice	District capitals	263	2.78	2.7	0.33	1.9	3.7	0.1949
	Smaller cities	242	2.78	2.8	0.36	1.4	3.9	
Control over practice	District capitals	263	3.00	3.0	0.41	1.8	4.0	0.0006
	Smaller cities	242	3.00	2.9	0.54	1.0	4.0	
Communication about patients	District capitals	263	3.00	3.0	0.43	1.8	4.0	0.0569
	Smaller cities	242	3.00	3.0	0.43	1.6	3.9	
Teamwork	District capitals	263	2.20	2.2	0.54	1.0	4.0	0.7687
	Smaller cities	242	2.20	2.2	0.58	1.0	3.6	
Handling disagreements	District capitals	263	2.50	2.6	0.46	1.3	3.8	0.1986
	Smaller cities	242	2.50	2.5	0.48	1.0	4.0	
Staff relationships	District capitals	263	3.00	3.0	0.45	2.0	4.0	0.2325
	Smaller cities	242	3.00	2.9	0.47	1.3	4.0	
Internal work motivation	District capitals	263	3.00	2.8	0.37	2.0	4.0	0.6686
	Smaller cities	242	3.00	2.8	0.47	1.0	4.0	
Cultural sensitivity	District capitals	263	3.00	2.7	0.51	1.0	4.0	0.0052
	Smaller cities	242	3.00	2.8	0.59	1.0	4.0	
RPPE total score	District capitals	263	2.79	2.8	0.26	2.1	3.4	0.1526
	Smaller cities	242	2.74	2.7	0.30	1.6	3.7	

**Table 4:** Differences in rating of the professional practice environment according to job position.

RPPE Scale Domain	Work Position	*n*	Median	Mean	SD	Min	Max	*p*-Value
Leadership and autonomy in clinical practice	General nurse	389	2.78	2.7	0.34	1.4	3.9	0.0443
	Practical nurse	112	2.83	2.8	0.36	1.8	3.9	
Control over practice	General nurse	389	3.00	3.0	0.49	1.0	4.0	0.1813
	Practical nurse	112	3.00	2.9	0.43	1.2	3.8	
Communication about patients	General nurse	389	3.00	3.0	0.43	1.6	4.0	0.0884
	Practical nurse	112	3.00	3.1	0.41	1.9	3.9	
Teamwork	General nurse	389	2.20	2.3	0.56	1.0	4.0	0.0175
	Practical nurse	112	2.00	2.1	0.51	1.0	3.6	
Handling disagreements	General nurse	389	2.75	2.6	0.47	1.3	4.0	0.0618
	Practical nurse	112	2.50	2.5	0.48	1.0	3.8	
Staff relationships	General nurse	389	3.00	3.0	0.45	1.3	4.0	0.0636
	Practical nurse	112	3.00	2.9	0.46	1.7	4.0	
Internal work motivation	General nurse	389	3.00	2.8	0.43	1.0	4.0	0.6804
	Practical nurse	112	3.00	2.8	0.38	2.0	4.0	
Cultural sensitivity	General nurse	389	3.00	2.8	0.54	1.0	4.0	0.2744
	Practical nurse	112	3.00	2.7	0.59	1.5	4.0	
RPPE total score	General nurse	389	2.77	2.8	0.29	1.6	3.7	0.522
	Practical nurse	112	2.74	2.8	0.25	2.1	3.4	

**Table 5:** Correlations between the practice environment and missed care and other variables.

	MISSCARE Survey Total Score	Satisfaction with Current Position	Satisfaction with Being a Nurse or a Nurse Assistant	Satisfaction with the Level of Teamwork on This Unit
MISSCARE Survey total score	1	−0.1308 *	−0.0875	−0.0688
RPPE total score	−0.2141 *	0.4879 *	0.2721 *	0.4576 *
Leadership and autonomy in clinical practice	−0.1044 *	0.2549 *	0.1502 *	0.4257 *
Control over practice	−0.1603 *	0.3920 *	0.1979 *	0.3408 *
Communication about patients	−0.0872	0.3749 *	0.2262 *	0.2556 *
Teamwork	−0.1753 *	0.4127 *	0.2242 *	0.2871 *
Handling disagreements	−0.1172 *	0.2104 *	0.0865	0.1677 *
Staff relationships	−0.2774 *	0.2160 *	0.2096 *	0.1926 *
Internal work motivation	−0.1343 *	0.3327 *	0.1965 *	0.3082 *
Cultural sensitivity	−0.1098 *	0.2922 *	0.1374 *	0.3352 *

* *p* < 0.05.

## References

[1] Zhao Y., Ma D., Wan Z., Sun D., Li H., Sun J., Jiao S. (2019). Associations between work environment and implicit rationing of nursing care: A systematic review. J. Nurs. Manag..

[2] Wiskow C., Albreht T., de Pietro C. (2010). How to Create an Attractive and Supportive Working Environment for Health Professionals. http://www.euro.who.int/__data/assets/pdf_file/0018/124416/e94293.pdf.

[3] Ausserhofer D., Zander B., Busse R., Schubert M., De Geest S., Rafferty A.M., Ball J., Scott P.A., Kinnunen J., Heinen M. (2013). Prevalence, patterns and predictors of nursing care left undone in European hospitals: Results from the multicountry cross-sectional RN4CAST study. BMJ Qual. Saf..

[4] Hessels A.J., Flynn L., Cimiotti J.P., Cadmus E., Gershon R.R. (2015). The Impact of the Nursing Practice Environment on Missed Nursing Care. Clin. Nurs. Stud..

[5] Zúñiga F., Ausserhofer D., Hamers J.P.H., Engberg S., Simon M., Schwendimann R. (2015). The relationship of staffing and work environment with implicit rationing of nursing care in Swiss nursing homes—A cross-sectional study. Int. J. Nurs. Stud..

[6] Aiken L., Sloane U.M., Clarke S., Poghosyan L., Cho E., You L., Finlayson M., Kanai-Pak M., Aungsuroch Y. (2011). Importance of work environments on hospital outcomes in nine countries. Int. J. Qual. Heal. Care.

[7] Nantsupawat A., Kunaviktikul W., Thienthong H., Poghosyan L., Nantsupawat R., Wichaikhum O.-A. (2016). Effects of nurse work environment on job dissatisfaction, burnout, intention to leave. Int. Nurs. Rev..

[8] Kanai-Pak M., Aiken L., Sloane D.M., Poghosyan L. (2008). Poor work environments and nurse inexperience are associated with burnout, job dissatisfaction and quality deficits in Japanese hospitals. J. Clin. Nurs..

[9] Lake E.T. (2002). Development of the practice environment scale of the nursing work index. Res. Nurs. Heal..

[10] Friese C., Lake E.T., Aiken L.H., Silber J.H., Sochalski J. (2008). Hospital Nurse Practice Environments and Outcomes for Surgical Oncology Patients. Heal. Serv. Res..

[11] Norman R.M., Sjetne I.S. (2017). Measuring nurses’ perception of work environment: A scoping review of questionnaires. BMC Nurs..

[12] Lake E., French R., O’Rourke K., Sanders J., Srinivas S.K. (2019). Linking the work environment to missed nursing care in labour and delivery. J. Nurs. Manag..

[13] Chen H.-L., Digiacomo M., Salamonson Y., Li Y., Huai B., Currow D.C. (2015). Nurses’ perceptions of their professional practice environment: A cross-sectional study. J. Clin. Nurs..

[14] Erickson J.I., Duffy M.E., Ditomassi M., Jones D. (2009). Psychometric Evaluation of the Revised Professional Practice Environment (RPPE) Scale. JONA J. Nurs. Adm..

[15] Papastavrou E., Acaroglu R., Şendir M., Berg A., Efstathiou G., Idvall E., Kalafati M., Katajisto J., Leino-Kilpi H., Lemonidou C. (2015). The relationship between individualized care and the practice environment: An international study. Int. J. Nurs. Stud..

[16] Suhonen R., Stolt M., Gustafsson M.-L., Katajisto J., Charalambous A. (2013). The associations among the ethical climate, the professional practice environment and individualized care in care settings for older people. J. Adv. Nurs..

[17] Lambrou P., Papastavrou E., Merkouris A., Middleton N. (2015). Professional environment and patient safety in emergency departments. Int. Emerg. Nurs..

[18] Page A.E.K. (2004). Transforming Nurses’ Work Environments to Improve Patient Safety: The Institute of Medicine Recommendations. Policy, Politi- Nurs. Pr..

[19] Kalisch B.J., Landstrom G., Williams R.A. (2009). Missed nursing care: Errors of omission. Nurs. Outlook.

[20] Kalisch B.J., Williams R.A. (2009). Development and Psychometric Testing of a Tool to Measure Missed Nursing Care. JONA J. Nurs. Adm..

[21] Kalisch B.J., Lee K.H. (2010). The impact of teamwork on missed nursing care. Nurs. Outlook.

[22] Kalisch B. (2016). Errors of Omission: How Missed Nursing Care Imperils Patients. J. Nurs. Regul..

[23] Zelenikova R., Gurková E., Jarošová D. (2019). Missed nursing care measured by MISSCARE Survey—The first pilot study in the Czech Republic and Slovakia. Central Eur. J. Nurs. Midwifery.

[24] Mueller C.W., McCloskey J.C. (1990). Nurses’ job satisfaction: A proposed measure. Nurs. Res..

[25] Roche M., Duffield C., Friedman S., Twigg D.E., Dimitrelis S., Rowbotham S. (2016). Changes to nurses’ practice environment over time. J. Nurs. Manag..

[26] Dorigan G.H., Guirardello E.D.B. (2017). Ambiente da prática, satisfação e clima de segurança: Percepção dos enfermeiros. Acta Paul. de Enferm..

[27] Jarošová D., Zelenikova R. (2019). Unfinished nursing care—The first pilot study in the Czech Republic. Kontakt.

[28] Aiken L.H., Clarke S., Sloane D.M., Sochalski J.A., Busse R., Clarke H., Giovannetti P., Hunt J., Rafferty A.M., Shamian J. (2001). Nurses’ Reports on Hospital Care in Five Countries. Heal. Aff..

[29] Kalisch B.J., Tschannen D., Lee K.H. (2011). Do staffing levels predict missed nursing care?. Int. J. Qual. Heal. Care.

[30] Siqueira L.D.C., Caliri M.H.L., Kalisch B., Dantas R.A.S. (2013). Cultural adaptation and internal consistency analysis of the MISSCARE Survey for use in Brazil. Rev. Latino-Americana de Enferm..

[31] Griffiths P., Maruotti A., Saucedo A.R., Redfern O., E Ball J., Briggs J., Dall’Ora C., E Schmidt P., Smith G.B., Bloor K. (2018). Nurse staffing, nursing assistants and hospital mortality: Retrospective longitudinal cohort study. BMJ Qual. Saf..

[32] Aiken L., Shang J., Xue Y., Sloane U.M. (2012). Hospital Use of Agency-Employed Supplemental Nurses and Patient Mortality and Failure to Rescue. Heal. Serv. Res..

[33] Lambrou P., Merkouris A., Middleton N., Papastavrou E. (2014). Nurses’ perception of their professional practice environment in relation to job satisfaction: A review of quantitative studies. Health Sci. J..

[34] Liu Y., Aungsuroch Y., Yunibhand J. (2015). Job satisfaction in nursing: A concept analysis study. Int. Nurs. Rev..

[35] Blackman I., Henderson J., Willis E., Hamilton P., Toffoli L., Verrall C., Abery E., Harvey C. (2014). Factors influencing why nursing care is missed. J. Clin. Nurs..

[36] Park S.H., Hanchett M., Ma C. (2018). Practice Environment Characteristics Associated With Missed Nursing Care. J. Nurs. Sch..

[37] Lake E., Riman K.A., Sloane D.M. (2020). Improved work environments and staffing lead to less missed nursing care: A panel study. J. Nurs. Manag..

[38] Kim K.-J., Yoo M.S., Seo E.J. (2018). Exploring the Influence of Nursing Work Environment and Patient Safety Culture on Missed Nursing Care in Korea. Asian Nurs. Res..

